# An integrated method for lightweight design and additive manufacturing of UAV arms

**DOI:** 10.1371/journal.pone.0344000

**Published:** 2026-03-11

**Authors:** Ruoyu Wang, Wenwei Yang, Guoying Pang, Zhiru Liu, Xue Rao, Yangyang Yu

**Affiliations:** 1 School of Intelligent Media and Design Art, Tianjin Renai College, Tianjin, China; 2 School of Mechanical and Power Engineering, Tianjin Renai College, Tianjin, China; 3 State Key Laboratory of Engine, Tianjin University, Tianjin, China; Beijing Institute of Technology, CHINA

## Abstract

Topology optimization and additive manufacturing (AM) have been widely applied to the lightweight design and fabrication of unmanned aerial vehicles (UAVs). However, existing topology optimization methods for UAVs typically assume isotropic materials, neglecting the anisotropy inherent in AM and the associated manufacturing precision constraints. This paper proposes a lightweight integrated method in MATLAB R2021a for the design and AM of UAV arms that simultaneously accounts for printing-induced anisotropy and minimum feature size constraints. A topology optimization model is proposed that uses nodal density and element printing angle as coupled design variables, and the corresponding sensitivity analysis is carried out. In the manufacturing phase, a contour-offset strategy is employed to generate printing paths for the optimized structures, achieving effective force transmission. The effects of manufacturing and optimization parameters on the design results are systematically investigated. The results show that, compared with the traditional optimization method, the compliance difference between the optimized structure obtained by the proposed method and the traditional method is only 0.46%. Furthermore, while ensuring manufacturability, printing efficiency is improved by approximately 69%. This approach establishes a unified design-to-manufacturing workflow, providing both a theoretical foundation and a practical pathway for the intelligent design and efficient fabrication of UAVs and other lightweight structural components.

## 1. Introduction

Unmanned Aerial Vehicles (UAVs) have rapidly developed and been widely applied in fields such as aerial photography, logistics, agricultural monitoring, and disaster rescue due to their advantages in flexibility, low operational costs, and high safety [1,2]. As the demand for extended flight time and increased payload capacity continues to rise, weight reduction has become a key strategy to improve UAV flight efficiency, manoeuvrability, and overall performance. UAV weight reduction can be achieved through two primary approaches: integrating lightweight electronic systems and designing lightweight structures. Among these, structural lightweight design directly affects the load-bearing system and is the fundamental strategy for enhancing overall mechanical performance and flight efficiency. At the same time, dynamically optimizing the drone’s trajectory to maximize flight time has also become an effective method [3,4].

Topology optimization, as an innovative and efficient structural lightweight design method, optimizes material distribution within a predefined design domain to achieve the optimal balance between structural performance and weight. It has been widely applied in fields such as aerospace, mechanical engineering, and architecture [5,6]. In recent years, numerous studies have applied topology optimization to the design of critical UAV structural components, significantly enhancing their structural efficiency and lightweight performance. Leon et al. [7] employed SolidWorks software and a generation-based design algorithm to perform topology optimization on the frame structure of a quadcopter UAV. Finite element analysis results indicated that the optimized UAV frame could support a weight of at least 0.9 kilograms. MohamedZain et al. [8] utilized 3DEXPERIENCE software to optimize four main components of the UAV frame: the center top cover, side top covers, middle cover, and arms. Through a trade-off study, they evaluated and compared the mass, displacement, and stress of the generated parts. Rayed et al. [9] applied the topology optimization module in ANSYS to improve the UAV structure, reducing its mass from 387.65g to 342.56g, and assessed its vibration and fatigue characteristics through simulation studies. Xiang et al. [10] conducted topology optimization and analysis of a quadcopter arm structure using the Optistruct platform in Inspire software. Nvss et al. [11] performed stress-based topology optimization of a quadcopter UAV’s structure using ANSYS software to achieve an optimal product design layout. Bay and Eryıldız [12] employed SolidWorks software to achieve topology optimization for three UAV frame designs, emphasizing the importance of stress distribution, displacement, mass reduction, and flow characteristics. Balayan et al. [13] proposed an optimal design method of a quadcopter chassis using generative design and lightweight materials to advance precision agriculture. However, topology optimization often yields relatively complex designs, and traditional manufacturing processes for these parts often lead to higher production costs.

Compared to traditional manufacturing processes, Additive Manufacturing (AM), a layer-by-layer fabrication technology, offers significant advantages including shorter production cycles, greater automation, greater manufacturing flexibility, and lower costs [14–16]. AM enables the production of complex structures that are difficult to achieve with conventional methods, significantly increasing design freedom and providing new possibilities for UAV structural design and fabrication. Relevant studies have demonstrated the potential of AM in the UAV field [17–19]. With AM technology, complex UAV structures, such as lightweight quadcopter frames, servo-motor tilt brackets, UAV brackets, and arms, can be successfully fabricated, showcasing the immense potential of combining topology-optimized design with AM [9,12,20–24].

However, AM is not a fully unconstrained manufacturing process, and the associated manufacturing constraints must be considered during the topology optimization of UAV structures. Liu et al. [22] used a robust topology optimization formulation in the UAV bracket design to control the minimum length scale ensuring the manufacturability of thin structural components and generated a series of designs with varying minimum structural component sizes. However, the significant mechanical anisotropic behavior caused by the AM process was overlooked. Both numerical calculations and experimental studies have shown that the printing path significantly affects the mechanical properties of 3D printed components. The printing path, aligned with the load transfer direction, has a positive impact on mechanical performance, particularly bending and tensile strength, enabling components to achieve higher load-bearing capacity [25–27]. Existing studies on topology optimization for UAV structures are mostly based on the assumption of isotropic materials and conducted using commercial software, without accounting for the anisotropic characteristics of the printing process in the optimization, which limits the effective utilization of AM technology’s advantages. Therefore, it is crucial to incorporate both the anisotropic characteristics of printing and the minimum feature size constraint into the UAV topology optimization process.

This study proposes a lightweight integrated method for design and AM in MATLAB R2021a that incorporates both printing-induced anisotropy and minimum feature size constraints and focuses on the UAV arm. A topology optimization model is established using nodal density and element-wise printing angle as design variables, structural compliance minimization as the objective function, and material volume fraction as the explicit constraint. Sensitivity analysis is conducted to facilitate the optimization process. To ensure the high performance of the manufactured structure, an automated manufacturing process is developed using a contour-offset path strategy. In addition, the influence of minimum feature size constraints, printing anisotropy, and initial design parameters on the optimization results is systematically investigated. The proposed method achieves an optimized UAV arm design that balances structural performance and printability, offering a novel approach to the intelligent design and efficient manufacturing of UAVs and other lightweight equipment.

## 2. Integrated optimization method for design and AM

**Fig 1:**
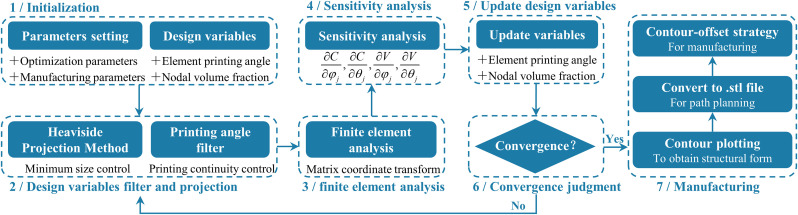
The flowchart of the proposed integrated optimization method.

The proposed method introduces two types of design variables and performs corresponding sensitivity analysis to obtain the final optimization results, thereby ensuring the manufacturability of the structure and the effective utilization of printing anisotropy. A contour-offset strategy is employed to maximize the benefits of the anisotropic properties introduced by AM. The flowchart of the proposed integrated optimization method is illustrated in Fig 1, and the detailed steps are as follows: (1) Initialization involves the selection of design variables, optimization parameters, and printing parameters; (2) Filtering and projection to enforce minimum feature size control and ensure continuity suitable for printing; (3) Finite element analysis to provide data for the subsequent sensitivity analysis; (4) Sensitivity analysis to guide the optimization of the design variables; (5) Design variable update using an optimization algorithm; (6) Convergence check and if the convergence condition is met, the structural form is finalized and the corresponding path planning is generated for AM. If not, the process returns to step (2) and iterates.

### 2.1. Optimization problem formulation

The method proposed in this study incorporates both printing-induced anisotropy and minimum feature size constraints. Each finite element *e* is given a printing angle *θ*_*e*_ and element node *i* is given a nodal volume fraction *φ*_*i*_. The minimum compliance optimization problem considering material volume, minimum feature size constraints, and printing anisotropy in this study is formulated as follows:


$$\begin{array}{c} find\;\;\varphi \text{=}{\left\{\varphi_{\text{1}},\varphi_{\text{2}},\ldots \;\ldots ,\varphi_{m}\right\}}^{T}\text{, }\theta \text{=}{\left\{\theta_{\text{1}},\theta_{\text{2}},\ldots \;\ldots ,\theta_{n}\right\}}^{T}\\ min\;\;C\left(\varphi ,\theta \right)=U{\left(\varphi ,\theta \right)}^{T}K\left(\varphi ,\theta \right)U\left(\varphi ,\theta \right)=\sum_{e=\text{1}}^{n}\rho_{e}{\left(\varphi \right)}^{q}u_{e}Tk_{e}\left(\theta_{e}\right)u_{e}\\ s.t.\;K\left(\varphi ,\theta \right)U\left(\varphi ,\theta \right)=F\\ \;\;V=\sum_{e=\text{1}}^{n}\rho_{e}\left(\varphi \right)v_{e}\leq fV_{\text{0}}\\ \;\;\varphi_{min}\leq \varphi_{i}\leq 1\text{,}\left(i=1,2,\ldots ,m\right)\\ \;{\text{-180}}^{\circ }\leq \theta_{e}\leq {\text{180}}^{\circ }\text{,}\left(e=1,2,\ldots ,n\right) \end{array}$$ (1)

where ***φ*** and ***θ*** are the design variables, which are nodal volume fraction and element printing angle vectors, respectively; *n* is the number of elements used to discretize the design domain and *m* is the number of element nodes; *C*(***φ***,***θ***) is the structural compliance, which is equal to the sum of the element strain energy; *ρ*_*e*_ and *v*_*e*_ are the density and volume of the *e*^th^ element, respectively; ***K***, ***F***, and ***U*** are the global stiffness matrix, global load, and nodal displacement vectors, respectively; *V* and *V*_0_ are the material and design domain volumes, respectively; and *f* is the prescribed volume fraction; *φ*_min_ is the minimum allowable nodal volume fraction and is set as 10^−8^; *q* is the penalization power; ***u***_*e*_ is the displacement vector associated with the *e*^th^ element; ***k***_*e*_(*θ*_*e*_) is the *e*^th^ element stiffness matrix of a solid element.

The equilibrium equation of the linear elastostatic problem is solved using the finite element method as ***K***(***φ***,***θ***)***U***(***φ***,***θ***)=***F***. ***K*** can be assembled by the element stiffness matrix ***k***_*e*_(*θ*_*e*_). Under these circumstances, the global stiffness matrix can be calculated as:


$$\begin{array}{c} K\left(\varphi ,\theta \right)\text{=}\sum_{e=\text{1}}^{n}\rho_{e}{\left(\varphi \right)}^{q}k_{e}\left(\theta_{e}\right)\\ k_{e}\left(\theta_{e}\right)\text{=}\int_{\Omega e}B^{T}D\left(\theta_{e}\right)Bd\Omega \end{array}$$ (2)

where ***K***(***φ***,***θ***) is the global stiffness matrix; $k_{e}\left(\theta_{e}\right)$ and $D\left(\theta_{e}\right)$ are the *e*^th^ element stiffness matrix and elastic matrix of a solid element, respectively, and they depend on the printing angle *θ*_*e*_ of the *e*^th^ element; *B* is the strain–displacement matrix; $\Omega_{e}$represents the region occupied by the *e*^th^ finite element; *q* is the penalization factor of stiffness; here, *q* is initialized at 1.0 and is increased by 0.5 if a change in the objective function has been reached 0.005, and the maximum value of *q* is set to 5.

A certain angle (*θ*_*e*_) exists between the local and global coordinate systems. After being rotated by *θ*_*e*_, the element elasticity matrix (***D***(*θ*_*e*_)) can be expressed as [28]:


$$D\left(\theta_{e}\right)=T\left(\theta e\right)D_{\text{0}}T{\left(\theta_{e}\right)}^{\text{T}}$$ (3)

where ***D***_0_ is the original elastic matrix under the local coordinate system, and ***T***(*θ*_*e*_) is the transform matrix, which is used to conduct the matrix coordinate transform. For the two-dimensional scenario, they are expressed as:


$$\begin{array}{c} D_{\text{0}}=\left[\begin{array}{ccc} @ccc@\frac{E_{\text{1}}}{1-\nu_{12}\nu_{21}} & \frac{\nu_{21}E_{\text{1}}}{1-\nu_{12}\nu_{21}} & 0\mathrm{\backslash vspace}2mm\\ \frac{\nu_{12}E_{\text{2}}}{1-\nu_{12}\nu_{21}} & \frac{E_{\text{2}}}{1-\nu_{12}\nu_{21}} & 0\mathrm{\backslash vspace}2mm\\ 0 & 0 & G_{12} \end{array}\right] \end{array}$$ (4)

$$\begin{array}{c} T\left(\theta_{e}\right)=\left[\begin{array}{ccc} @ccc@{cos}^{\text{2}}\theta_{e} & {sin}^{\text{2}}\theta_{e} & -2sin\theta_{e}cos\theta_{e}\\ {sin}^{\text{2}}\theta_{e} & {cos}^{\text{2}}\theta_{e} & 2sin\theta_{e}cos\theta_{e}\\ sin\theta_{e}cos\theta_{e} & -sin\theta_{e}cos\theta_{e} & {cos}^{\text{2}}\theta_{e}-{sin}^{\text{2}}\theta_{e} \end{array}\right] \end{array}$$ (5)

where *E*_1_ and *E*_2_ are the Young’s modulus in the 1st and 2nd principal elastic directions; *G*_12_ is the shear modulus in the 1–2 plane, which is the printing plane; *ν*_12_ and *ν*_21_ are the Poisson’s ratios, which are related through *ν*_12_/*E*_1_ = *ν*_21_/*E*_2_.

The printing angle filter is adopted to ensure continuity, which is important not only for manufacturing issues but also to avoid stress concentrations at discontinuous paths. In this work, the continuity is controlled by using the density filtering proposed by Bruns and Tortorelli [29]. The density filter causes the original variables to loose their physical meaning, so the filtered variables are used as the solution to the optimization problem rather than the original variables.

Before performing finite element analysis, the orientation design variables of the central element are corrected by using the orientation design variables of all elements within the filtering range. That is, the orientation of the central element is replaced by the weighted average of the orientation angles of each element within the filtering radius. Hence, the filtered variable for the element *e* can be expressed as:


$${\overset{―}{\theta }}_{e}=\frac{\text{1}}{\sum_{i\in \Omega_{e}}H_{ei}}\sum_{i\in \Omega_{e}}H_{ei}\theta_{i}$$ (6)

where ${\overset{―}{\theta }}_{e}$ is the filtered orientation variable of *e*^th^ element; $\theta_{i}$ is the original orientation variable, which is assigned to the centroid of element *i*. The neighborhood of element *e*, here termed Ω_*e*_, is generally specified by the elements that have centers within a particular filter radius (*r*_min1_) of the center of element *e*; *H*_*ei*_ is a weight factor defined as *H*_*ei*_ = max (0, *r*_min1_-△(*e*, *i*)), where △(*e*, *i*) is the center-to-center distance of elements *i* and *e*.

The minimum length scale of structural features is controlled by the Heaviside Projection Method (HPM) [30,31]. In HPM, the design variables *φ*_*i*_ are associated with a material phase and projected onto the finite elements by a Heaviside function. The design variables *φ*_*i*_ are first mapped onto element space by computing the weighted average of design variables in set *N*_*e*_ for each element. The weighted average *μ*_*e*_(***φ***) is expressed as:


$$\mu_{e}\left(\varphi \right)=\frac{\sum_{i\in N_{e}}\varphi_{i}w({𝐱}_{i}-{\overset{―}{𝐱}}^{e})}{\sum_{i\in N_{e}}w({𝐱}_{i}-{\overset{―}{𝐱}}^{e})}$$ (7)

where *N*_*e*_ is the set of nodes in the domain of influence of element *e*; **x**_*i*_ is the position of node *j*; $w({𝐱}_{i}-{\overset{―}{𝐱}}^{e})$ is a linear weighting function defined as:


$$w({𝐱}_{i}-{\overset{―}{𝐱}}^{e})\text{=}\left\{\begin{array}{c} @c@\left(r_{min2}-\|{𝐱}_{i}-{\overset{―}{𝐱}}^{e}\|\right)\;/r_{min2}\\ 0 \end{array}\;\begin{array}{c} \text{if }{𝐱}_{i}\in N_{e}\\ \text{otherwise} \end{array}\right\}$$ (8)

where *r*_min2_ is the filter radius of the size constraint. Then, the regularized Heaviside function relates the weighted average *μ*_*e*_(***φ***) to element relative densities *ρ*_*e*_ through the following equation:


$$\rho_{e}=1-e^{-\beta \mu_{e}\left(\varphi \right)}+\mu_{e}\left(\varphi \right)e^{-\beta }$$ (9)

where *β* is the curvature of the regularization that approaches the Heaviside function as *β* approaches infinity. A continuation method is used, with *β* initially set to a small value and increased in subsequent iterations to achieve a near 0–1 topology.

### 2.2. Sensitivity analysis and optimization algorithm

Different from traditional topology optimization design methods, due to the introduction of new design variables and constraints, corresponding formula derivation is required. The sensitivities of the compliance with respect to design variables are calculated using the chain rule as follows:


$$\begin{array}{c} \frac{\partial C}{\partial \varphi_{j}}=\sum_{e\in N_{j}}\frac{\partial C}{\partial \rho_{e}}\frac{\partial \rho_{e}}{\partial \mu_{e}}\frac{\partial \mu_{e}}{\partial \varphi_{j}}\\ \frac{\partial C}{\partial \theta_{j}}=\sum_{e\in \Omega_{j}}\frac{\partial C}{\partial {\overset{―}{\theta }}_{e}}\frac{\partial {\overset{―}{\theta }}_{e}}{\partial \theta_{j}}=\sum_{e\in \Omega_{j}}\frac{\text{1}}{\sum_{i\in \Omega_{e}}H_{ei}}H_{je}\frac{\partial C}{\partial {\overset{―}{\theta }}_{e}} \end{array}$$ (10)

The following expression is obtained:


$$\begin{array}{c} \frac{\partial C}{\partial \rho_{e}}=-q{\rho_{e}}^{q-1}u_{e}Tk_{e}\left({\overset{―}{\theta }}_{e}\right)u_{e}\\ \frac{\partial C}{\partial {\tilde{\theta }}_{e}}=-{\rho_{e}}^{q}u_{e}T\frac{\partial k_{e}\left({\overset{―}{\theta }}_{e}\right)}{\partial {\overset{―}{\theta }}_{e}}u_{e}\text{=}-{\rho_{e}}^{q}u_{e}T\int_{\Omega e}B^{T}\frac{\partial D\left({\overset{―}{\theta }}_{e}\right)}{\partial {\overset{―}{\theta }}_{e}}Bd\Omega u_{e}\\ \frac{\partial \rho_{e}}{\partial \mu_{e}}\text{=}\beta e^{-\beta \mu_{e}}+e^{-\beta }\\ \frac{\partial \mu_{e}}{\partial \varphi_{j}}=\frac{w({𝐱}_{j}-{\overset{―}{𝐱}}^{e})}{\sum_{j\in N_{e}}w({𝐱}_{j}-{\overset{―}{𝐱}}^{e})} \end{array}$$ (11)

Then, the derivative $\partial D\left({\overset{―}{\theta }}_{e}\right)/\partial {\overset{―}{\theta }}_{e}$ is obtained based on Eq. (3):


$$\frac{\partial D\left({\overset{―}{\theta }}_{e}\right)}{\partial {\overset{―}{\theta }}_{e}}\text{=}\frac{\partial T\left({\overset{―}{\theta }}_{e}\right)}{\partial {\overset{―}{\theta }}_{e}}D_{\text{0}}T{\left({\overset{―}{\theta }}_{e}\right)}^{\text{T}}\text{+}T\left({\overset{―}{\theta }}_{e}\right)D_{\text{0}}\frac{\partial T{\left({\overset{―}{\theta }}_{e}\right)}^{T}}{\partial {\overset{―}{\theta }}_{e}}$$ (12)

According to the definition in Eq. (1), the sensitivity of the volume constraint with respect to design variables is:


$$\begin{array}{c} \frac{\partial V}{\partial \varphi_{j}}=\sum_{e\in N_{j}}\frac{\partial V}{\partial \rho_{e}}\frac{\partial \rho_{e}}{\partial \mu_{e}}\frac{\partial \mu_{e}}{\partial \varphi_{j}}=\sum_{e\in N_{j}}v_{e}\frac{\partial \rho_{e}}{\partial \mu_{e}}\frac{\partial \mu_{e}}{\partial \varphi_{j}}\\ \frac{\partial V}{\partial \theta_{j}}=0 \end{array}$$ (13)

Based on the above sensitivity results, the design variables are updated using the Method of Moving Asymptotes (MMA) optimization scheme introduced by Svanberg [32]. The processes, including design variables filter and projection, finite element analysis, computation of objective and constraint values, sensitivity analysis, and updating design variables, are repeated until the solution is stably converged. The following condition is used to check the convergence:


$$\left|\frac{C^{I}-C^{I-1}}{C^{I-1}}\right|\leq \varepsilon$$ (14)

where *C*^*I*^ and *C*^*I*-1^ are objective values corresponding to the *I*^th^ and (*I*-1)^th^ iterations, respectively, and *ɛ* is the allowed convergence error.

## 3. Results and discussion

**Fig 2:**
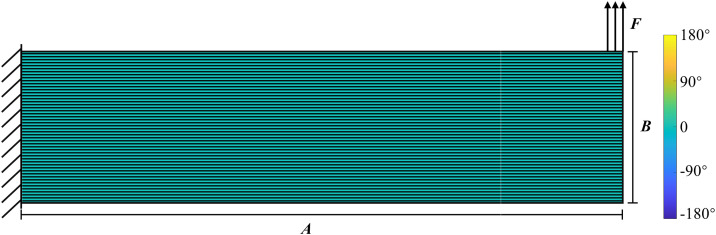
Design domain for UAV arm optimization.

The UAV arm optimization problem is illustrated in Fig 2. The design domain is discretized into a 200 × 50 grid of quadrilateral elements. A fixed boundary condition is applied at the left end, and a unit load (with a total magnitude of 1) is applied to five consecutive nodes at the upper-right corner. The material properties are *E*_1_ = 1, *E*_2_ = 0.5, *v*_12_ = 0.3, *G*_12_ = *E*_1_/(2 × (1 + *v*_12_)), *v*_21_ = *E*_2_ × *v*_12_*/E*_1_. The volume fraction is 0.5. The initial design assigns a nodal density of 1 and sets the corresponding element-wise printing angle to 0. Moreover, we adopt move limits to restrict the maximum allowable change in a design variable to avoid oscillation in the objective value during the iterative process. The parameters of the move limits are set as 0.1 and 2π/9, respectively. The minimum feature size constraint is set to four times the element size.

**Fig 3:**
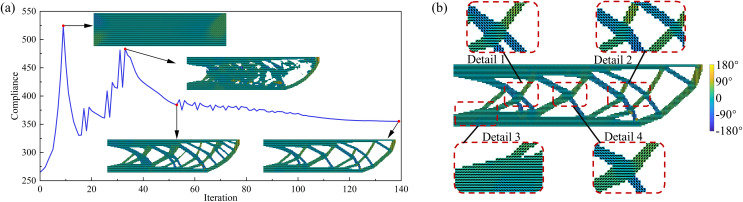
Optimization results of the UAV arm using the proposed method (a) convergence history of the objective function; (b) final optimized structure.

The UAV arm optimization results obtained using the proposed method are shown in Fig 3. Fig 3a presents the objective function convergence curve. As the optimization progresses, the compliance initially increases and then decreases, indicating the search for an improved material layout. The optimization converges after 139 iterations, yielding a design that satisfies both convergence criteria and design requirements. Because the final optimized results still contain intermediate elements, a threshold value of 0.3 is adopted to ensure a clear black–and–white representation of the structure. Specifically, elements with a density below 0.3 are set to 0, while those with a density of 0.3 or higher are set to 1. Accordingly, in this study, the final compliance objective function is evaluated based on the binarized element density distribution. The compliance increases from 265.03 to 351.06, while the volume fraction decreases by about 50%, which represents only a 32.46% increase in the objective function for a significant reduction in material usage. Additionally, as the iterations proceed, slender members that violate the minimum feature size constraint are progressively eliminated. At the same time, the element-wise printing angles, which were initially set to 0°, gradually evolve toward optimal orientations. The final optimized result is shown in Fig 3b, where the internal structure satisfies the minimum feature size constraint and the printing angles are aligned with the structure’s primary load paths. This alignment of the optimized printing angles with the structural force flow suggests that the contour-offset printing path strategy should be adopted in practice to effectively leverage the anisotropic mechanical advantages offered by the manufacturing process.

**Fig 4:**
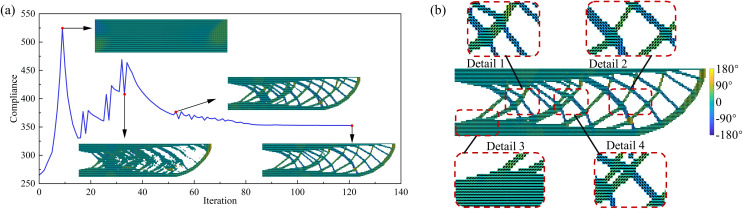
Optimization results of the UAV arm without considering minimum feature size constraint (a) convergence history of the objective function; (b) final optimized structure.

The optimization results of the UAV arm without considering the minimum feature size constraint are shown in Fig 4. Fig 4a presents the objective function convergence curve, and the final optimized result is shown in Fig 4b. As shown in Fig 4, during the optimization process, the element-wise printing angle gradually evolves from 0° to align with the structure’s primary load path. The final objective function value is 352.68, which is 1.62 lower than the proposed method’s objective function value, a decrease of about 0.46%, indicating that imposing the size constraint does not result in a loss of structural stiffness. However, without the minimum feature size constraint, the optimized structure contains many slender members and excessive intersecting branches, which could lead to printing accuracy issues, resulting in either an unprintable structure or poor quality. This further validates the necessity of incorporating size constraints in the UAV arm optimization problem.

**Fig 5:**
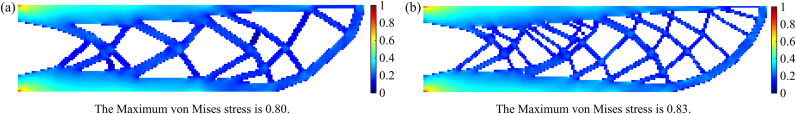
The von Mises stress distribution of the UAV arm (a) with minimum feature size constraint; (b) without minimum feature size constraint.

To further compare the optimization results with and without considering the size constraint, the corresponding von Mises stress distribution is shown in Fig 5. As shown in Fig 5, the maximum stress area is distributed at the left boundary, and the stress in the node area where the branches intersect is slightly greater than that in the surrounding area, which is caused by anisotropy. At the same time, after the size constraint is introduced, the maximum stress is reduced by 0.3, indicating that structural performance is not significantly reduced while ensuring manufacturability.

**Fig 6:**
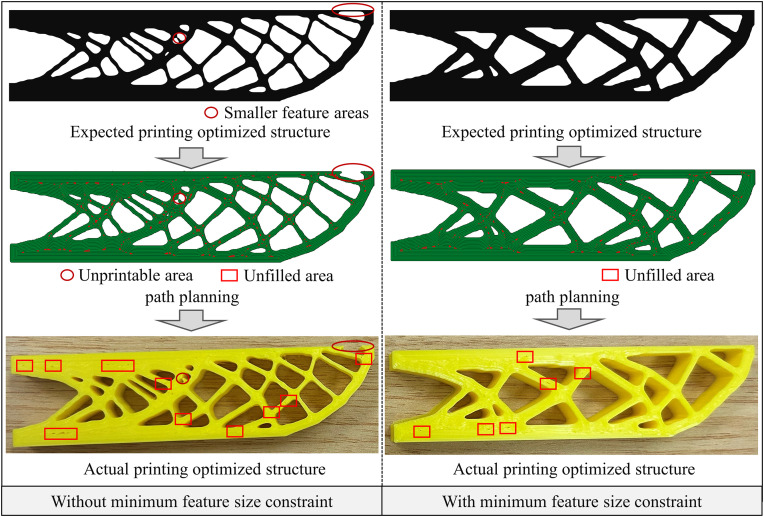
The printing results comparison of the proposed method and the traditional method.

Fig 6 compares the printing results obtained using the proposed method with those from the traditional method optimized without size constraints. The latter exhibits numerous unprintable and unfilled regions, hindering the consistency between design and manufacturing. Moreover, the resulting component is not manufacturable and fails to meet practical application requirements. In contrast, the proposed method effectively eliminates small feature regions, thereby ensuring manufacturability and achieving high-quality printing and filling. Table 1 summarizes the data comparison of printed results. The proposed method significantly reduces unprintable areas, reduces unfilled areas by approximately 32%, and shortens total printing time by about 8%. Furthermore, by reducing the presence of small features, the overall structural complexity and number of branches are minimized, leading to a 21% reduction in idle motion. Although decreasing the nozzle diameter can improve manufacturability, it results in a prolonged printing time of 2 hours and 59 minutes. In comparison, the proposed method requires only 56 minutes and 9 seconds, enhancing printing efficiency by approximately 69% while maintaining manufacturability.

**Table 1:** Data comparison of printed results.

Different methods	Number of unprintable areas	Number of unfilled areas	Pixels of unfilled areas	Model printing time	Idle time
Traditional method	2	355	4401	1h1min	13min43s
Proposed method	0	270	2992	56min9s	11min4s

### 3.1. Effect of manufacturing parameters

#### 3.1.1. Different feature minimum size constraints.

**Fig 7:**
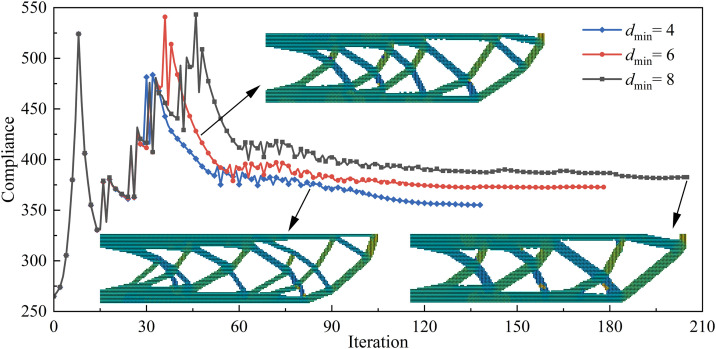
Objective function iteration curves for different minimum feature size constraints.

The minimum feature size constraint, as a key parameter controlling the structural detail scale, directly influences the manufacturability and structural continuity of the optimized design. To obtain more design results for the UAV arm structure and validate the effectiveness of the proposed method, optimization designs with different minimum feature size constraints were performed. The objective function iteration curves for minimum feature size constraints of 4, 6, and 8 are shown in Fig 7. The initial objective function value was 265.03, while the final objective function values were 351.06, 353.09, and 351.13, respectively. With the volume fraction reduced by approximately 50%, the corresponding increases in the objective function were 32.46%, 33.23%, and 32.49%. A comparative analysis indicates that increasing the minimum size constraint leads to a monotonic increase in the objective function; however, the rate of increase remains relatively low. Notably, when the size constraint is doubled, the resulting difference in the objective function value is only 0.07, demonstrating a limited sensitivity of the optimization outcome to the minimum size constraint.

**Fig 8:**
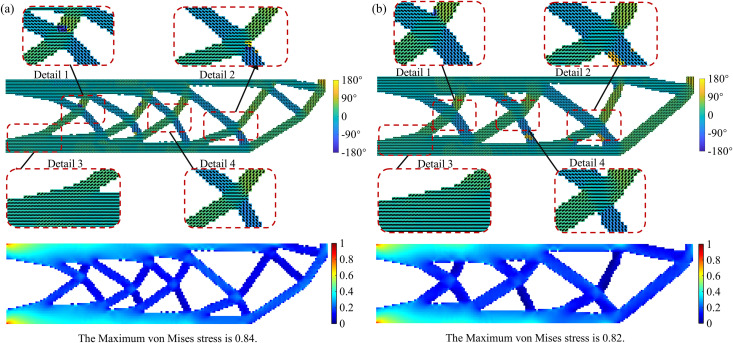
Optimization results with minimum feature size constraints (a) 6; (b) 8.

The optimization results with different minimum feature size constraints are shown in Fig 8. The results indicate that smaller minimum feature size constraints allow the formation of more detailed structures, which enhance local load-bearing capacity but may pose manufacturing difficulties. As the minimum feature size constraint increases, simpler and smoother structures are more easily formed, improving manufacturing robustness, but possibly sacrificing some performance. Meanwhile, the element-wise printing angles of the optimized components ensure good continuity and align with the structural load path, effectively exploiting the printing anisotropy and further validating the applicability of the proposed method.

#### 3.1.2. Different degrees of anisotropy.

**Fig 9:**
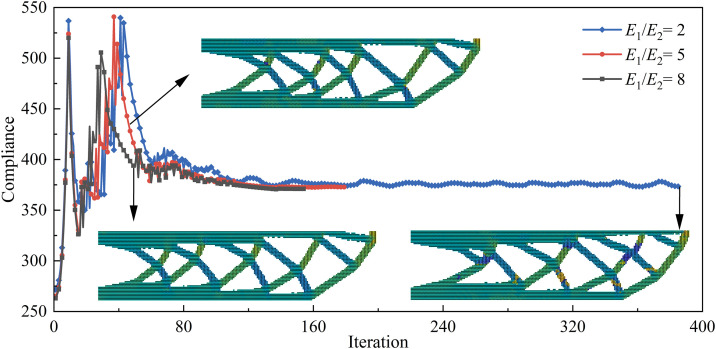
Objective function iteration curves for different degrees of anisotropy.

Due to the layering characteristics of AM, which lead to significant printing anisotropy, this section introduces different degrees of anisotropy (*E*_1_/*E*_2_ = 2, 5, and 8) for topology optimization analysis, with a minimum feature size set to 6. Fig 9 shows the objective function iteration curves for different degrees of anisotropy. From the convergence behavior of the objective function during the topology optimization process, it can be seen that as the number of iterations increases, the objective function value gradually stabilizes for each case, indicating that the optimization process is stable and convergent, and that feasible solutions satisfying the constraint conditions can be generated. Furthermore, as the degree of anisotropy increases, the objective function decreases.

**Fig 10:**
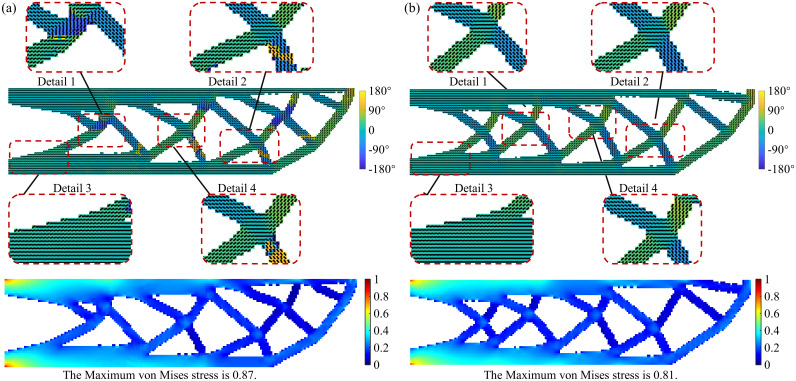
Optimized design results for different degrees of anisotropy (a) *E*_1_/*E*_2_ = 2; (b) *E*_1_/*E*_2_ = 8.

Fig 10 presents the optimized design results under different levels of material anisotropy, which reveals that the degree of anisotropy significantly influences the structural layout. Under conditions of high anisotropy, the internal features of the structure appear more ordered and regular. Meanwhile, as anisotropy increases, the distribution of element printing angles becomes more directional, aligning more closely with the primary load paths. Therefore, appropriately guiding and incorporating the anisotropic characteristics of the printing process during optimization can maximize structural performance.

### 3.2. Effect of optimization parameters

#### 3.2.1. Different volume fractions.

**Fig 11:**
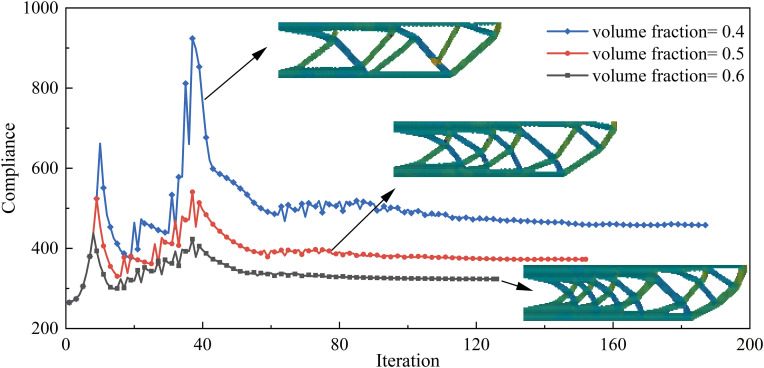
Objective function convergence curves under different volume fractions.

The volume fraction is a key control parameter in topology optimization, directly influencing the lightweight level of the optimized structure. Fig 11 shows the compliance evolution curves under different volume fractions. By comparing the compliance results of the optimized UAV arm structures under varying volume fractions, it can be observed that as the volume fraction decreases, the structural mass is significantly reduced, but the load-bearing capacity also declines. For volume fractions of 0.4, 0.5, 0.6, and 1, the corresponding objective function values are 418.77, 351.06, 309.98, and 265.03, respectively. Compared with the volume fractions of 1, the objective function increases by 58.01%, 32.46%, and 16.96%, respectively. These results indicate that the optimized UAV arm exhibits a higher load-bearing efficiency per unit weight. Consequently, the volume fraction can be selected according to specific application requirements to achieve tailored trade-offs between lightweight design and structural performance.

**Fig 12:**
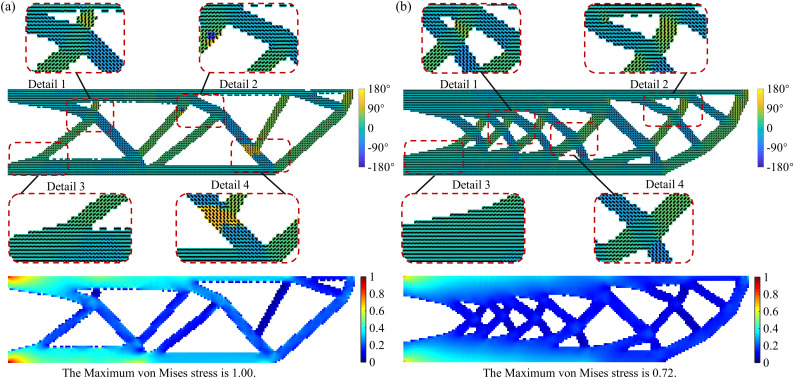
Optimization results under different volume fractions (a) 0.4; (b) 0.6.

Fig 12 shows the optimization results under different volume fractions. As the volume fraction decreases, the material distribution becomes sparser and more concentrated, with redundant material in non-critical areas gradually removed. Only the main load paths and key load-bearing regions are retained, resulting in a more streamlined and efficient topology. Additionally, the distribution of printing directions under various volume fractions shows that the element-wise printing angles tend to align with the main load paths. This alignment between printing directions and primary load direction effectively leverages the anisotropic properties of AM to enhance structural stiffness.

#### 3.2.2. Different initial aspect ratios.

**Fig 13:**
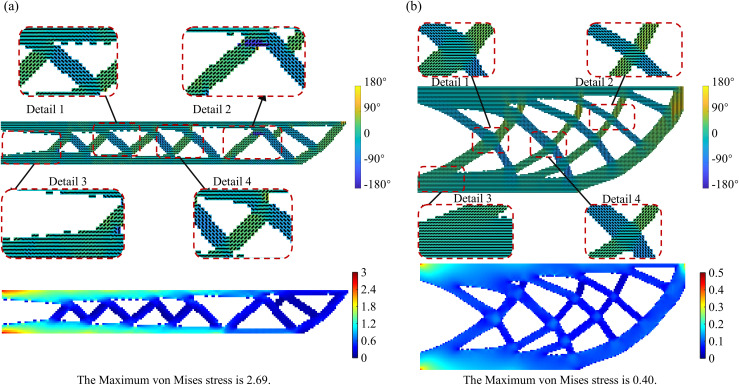
Optimization results under different initial aspect ratios (a) 200: 25; (b) 200: 80.

To study the impact of the initial aspect ratio on topology optimization results, this section compares design domains with aspect ratios of 200: 25 and 200: 80, while keeping the load and boundary conditions consistent. Fig 13 shows the optimization results under different initial aspect ratios. It can be observed that the element-wise printing angle shows significant differences across varying aspect ratios. In high aspect ratio cases, the printing direction aligns more consistently along the structural main axis, closely matching the force transmission path. This results in better continuity of printing angles, effectively enhancing the structure’s stiffness and mechanical performance. In contrast, under low aspect ratio conditions, the structure tends to spread more widely, leading to poorer continuity of the printing angles. In some areas, the printing angle deviates from the force transmission path, potentially reducing local stiffness or causing manufacturing issues. Therefore, selecting an appropriate aspect ratio not only influences structural topology optimization but also significantly affects the quality of the structure’s formation during the manufacturing process.

The aspect ratio of the initial design domain significantly affects the structural compliance in the optimization results. A larger aspect ratio results in a more elongated structure, which, although facilitating unidirectional force transfer, tends to reduce stiffness, as reflected by increased compliance. In contrast, a wider design domain helps guide forces along multiple secondary paths within the structure, distributing the load over a larger area and thereby improving overall stiffness. Therefore, the choice of aspect ratio not only influences the evolution of the structure’s form but also directly impacts the final mechanical performance. Properly selecting the geometric ratio of the UAV arm design domain is a critical prerequisite for achieving a balance between lightweight and stiffness performance.

#### 3.2.3. Different numbers of elements.

**Fig 14:**
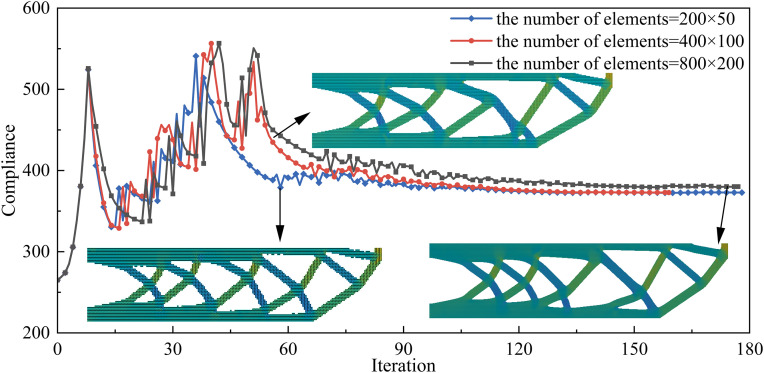
Objective function convergence curves under different numbers of elements.

To study the impact of different numbers of elements on structural performance and computational cost, this section compares numbers of elements with 200 × 50, 400 × 100 and 800 × 200, while keeping the load and boundary conditions consistent. Fig 14 shows the optimization results under numbers of elements. As the number of elements increases, the optimization iterations eventually converge, and the printing angle remains highly consistent with the structural members, verifying the effectiveness of the proposed method across different element numbers. Meanwhile, the minimum feature size constraint is satisfied for all cases with different numbers of elements, and no slender or fine-scale branches are observed in the optimized results. It is worth noting that due to the interaction between the node density and printing angle, the structural form differs with different element numbers, but the main force transmission path remains essentially consistent. Meanwhile, the structural performance and computational cost under different element numbers are compared in Table 2. The comparison shows that structural compliance differs little with different element numbers, but a fourfold increase in the element number corresponds to an order-of-magnitude increase in runtime.

**Table 2:** Structural performance and computational cost with different numbers of elements.

Numbers of elements	200 × 50	400 × 100	800 × 200
**Structural compliance**	351.30	350.90	349.88
**Computational cost**	449.65s	4391.66s	65568.15s

### 3.3. Manufacturing of the optimized UAV arm

Materials such as polylactic acid and acrylonitrile-butadiene-styrene (ABS) are commonly used for 3D printing topology-optimized UAV structures. A desktop-level printer based on the FDM principle offers good forming accuracy and stability. During printing, the equipment melts the thermoplastic material using a heated nozzle and builds up layer by layer along the set path, accurately reproducing the complex geometric features of the design model.

This section further explores the manufacturability of the UAV arm design. The method proposed in this paper effectively controls the minimum feature size constraint, ensuring that no thin areas in the optimized design are unprintable. As a result, all designs can be successfully manufactured. In the optimization process, the element-wise printing angles are adjusted along the force transmission path, ensuring that the printing direction aligns with the orientation of the structural members. This characteristic enables the use of a contour-offset strategy, which not only guarantees structural performance but also enables efficient and high-quality printing.

**Fig 15:**
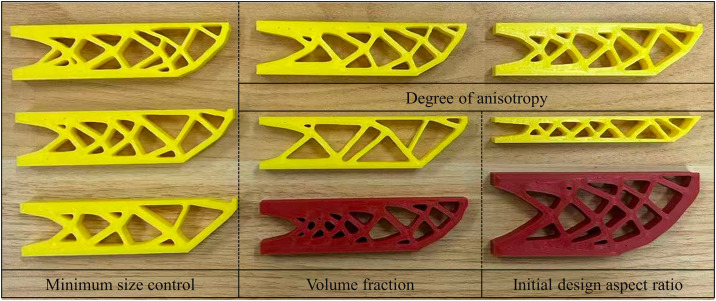
Summary of different design printing paths.

In the manufacturing process, the arm structure is set to a length of 100 mm, with printing parameter settings shown in Table 3. After slicing the model based on the topology optimization results, Fig 15 shows the AM path planning generated from the optimized design. As shown in Fig 15, the generated printing paths effectively reflect the rational distribution of materials and the main load-bearing paths of the structure, efficiently utilizing printing anisotropy to enhance the mechanical performance of the printed product.

**Table 3:** Printing parameter settings.

Layer thickness	Speed	Temperature	Material	Nozzle diameter	Path planning
0.2 mm	50 mm/s	230°C	ABS	0.4 mm	Contour offset

**Fig 16:**
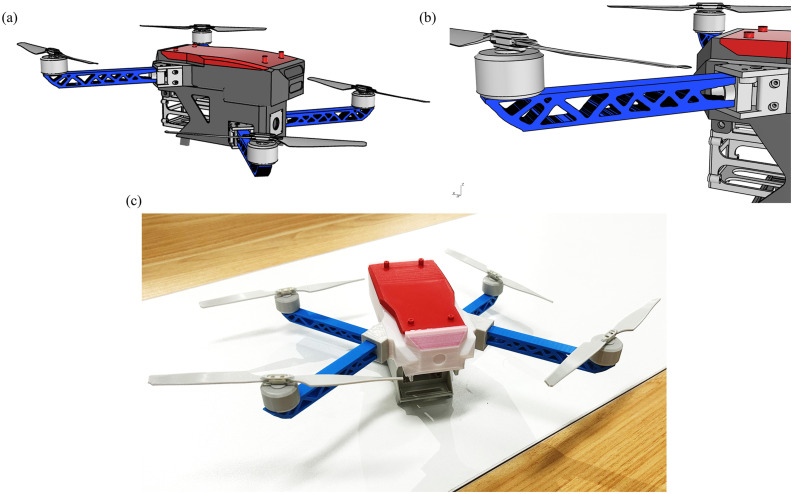
Assembled optimized arm onto UAV product (a) overall demonstration; (b) local demonstration; (c) final 3d printed result.

Fig 16a and b illustrate the concept of integrating the optimized arm design into the UAV product, providing a clear visualization of the optimized structural layout and adaptability in the actual product. Fig 16c shows the final 3D printed sample. The high-quality print with a clear structural outline, minimal warping, and no visible layering defects validates the rationality of the printing parameter settings and the effectiveness of the printing path planning. This printed sample provides a reliable physical basis for subsequent experimental tests and demonstrates the good engineering feasibility of this method.

## 4. Conclusions

This study proposed an integrated optimization method for lightweight design and AM that accounts for printing anisotropy and minimum feature size constraints. The effectiveness of the optimization method was verified using the UAV arms as an example. A series of structural optimization solutions was generated by considering multiple factors, including minimum feature size constraints, degrees of printing anisotropy, material volume fraction, and the aspect ratio of the initial design domain. Additionally, manufacturability was verified using a contour offset strategy. The results indicate that this method not only effectively ensures the stiffness and alignment of the load transfer path but also significantly enhances the feasibility and precision of the optimized results in the manufacturing process, validating the effectiveness of the proposed method in achieving dual optimization of both performance and manufacturability. The proposed method offers a theoretical basis and practical guidance for advancing intelligent structural design and AM of drones and other lightweight equipment.

Future research can further explore topology optimization of three-dimensional UAV structures while incorporating more complex manufacturing constraints, such as multi-material printing, thermomechanical performance, and residual stresses. Meanwhile, experimental validation is required to verify the effectiveness of the proposed optimization method. In addition, the application of multi-objective optimization strategies will become increasingly important, particularly for the simultaneous optimization of structural performance, stiffness-to-weight ratio, fatigue life, and manufacturability to achieve more comprehensive design objectives.

## Supporting information

S1 File Matlab codes of an integrated method for lightweight design and additive manufacturing of UAV arms. (DOCX)
