# Supplementary material for: An integrated method for lightweight design and additive manufacturing of UAV arms
Source: PLoS One. 2026 Mar 11;21(3):e0344000. doi: 10.1371/journal.pone.0344000 (PMC12978453; doi:10.1371/journal.pone.0344000)
Supplement: S1 File — (DOCX) [file pone.0344000.s001.docx]

function alltopominisizedouble(nelx,nely,volfrac,p1,rmin,eta,move)

clear all; clc; close all;

warning('off');

aa=200;

b=50;

nelx=200;

nely=nelx*b/aa;

ab=aa/nelx;

volfrac=0.5;

move=0.1;

movetheta=40*pi/180;

p1=1;

rmin=3;

rmin2=2;

ft=2;

fj=2

E1 = 1;

E2 = 0.5*E1;

NU12 =0.3;

G12=E1/(2*(1+NU12));

NU21=E2*NU12/E1;

thetamin=-2*pi;

thetamax=2*pi-10^(-8);

[KE,dKE,D0,trsT,dtrsT,B]=elementfea(E1,E2,G12,NU12,NU21,ab);

syms theta

B=double(B);

Ke=reshape(KE,1,64);

f = matlabFunction(Ke);

dKe=reshape(dKE,1,64);

f1 = matlabFunction(dKe);

TrsT=reshape(trsT,1,9);

f2 = matlabFunction(TrsT);

dTrsT=reshape(dtrsT,1,9);

f3 = matlabFunction(dTrsT);

nodenrs = reshape(1:(1+nelx)*(1+nely),1+nely,1+nelx);

edofVec = reshape(2*nodenrs(1:end-1,1:end-1)+1,nelx*nely,1);

edofMat = repmat(edofVec,1,8)+repmat([0 1 2*nely+[2 3 0 1] -2 -1],nelx*nely,1);

iK = reshape(kron(edofMat,ones(8,1))',64*nelx*nely,1);

jK = reshape(kron(edofMat,ones(1,8))',64*nelx*nely,1);

F = sparse(2*((nely+1)*nelx+1),1,1,2*(nely+1)*(nelx+1),1);

F = sparse(2*(nely+1)*(nelx+1),1);

F(2*((nely+1)*nelx+1):-2*(nely+1):2*((nely+1)*nelx+1)-2*4*(nely+1)/ab)=1/(4/ab+1);

fixeddofs = (1:2*(nely+1));

U = zeros(2*(nely+1)*(nelx+1),1);

alldofs = [1:2*(nely+1)*(nelx+1)];

freedofs = setdiff(alldofs,fixeddofs);

AllDomain=reshape([1:(nely+1)*(nelx+1)],nely+1,nelx+1);

ValidDomain=AllDomain;

PassDomain=[];

ValidDomain=setdiff(AllDomain,PassDomain);

AllDomain1=reshape([1:nely*nelx],nely,nelx);

ValidDomain1=AllDomain1;

PassDomain1=[];

ValidDomain1=setdiff(AllDomain1,PassDomain1);

iH = [];

jH = [];

sH = [];

k = 0;

for i1=1:nely

for j1=1:nelx

e1 = (j1-1)*nely+i1;

for m1=max(i1-(ceil(rmin/ab)-1),1):min(i1+1+(ceil(rmin/ab)-1),nely+1)

for n1=max(j1-(ceil(rmin/ab)-1),1):min(j1+1+(ceil(rmin/ab)-1),nelx+1)

e2 = (n1-1)*(nely+1)+m1;

k = k+1;

iH(k) = e1;

jH(k) = e2;

sH(k) = max(0,rmin-sqrt(((0.5+j1-1-n1+1)*ab)^2+((0.5+i1-1-m1+1)*ab)^2))/rmin;

end

end

end

end

H = sparse(iH,jH,sH);

Hs = sum(H,2);

Hsd = sum(H);

iH = ones(nelx*nely*(2*(ceil(rmin2/ab)-1)+1)^2,1);

jH = ones(size(iH));

sH = zeros(size(iH));

k = 0;

for i1 = 1:nelx

for j1 = 1:nely

e1 = (i1-1)*nely+j1;

for i2 = max(i1-(ceil(rmin2/ab)-1),1):min(i1+(ceil(rmin2/ab)-1),nelx)

for j2 = max(j1-(ceil(rmin2/ab)-1),1):min(j1+(ceil(rmin2/ab)-1),nely)

e2 = (i2-1)*nely+j2;

k = k+1;

iH(k) = e1;

jH(k) = e2;

d1=(i1-i2)*ab;

d2=(j2-j1)*ab;

sH(k) = max(0,rmin2-sqrt(d1^2+d2^2));

end

end

end

end

H2 = sparse(iH,jH,sH);

H2(PassDomain(:),:)=[];

H2(:,PassDomain(:))=[];

Hs2 = sum(H2,2);

x =ones(nely+1,nelx+1);

theta=0*ones(nely,nelx);

beta =1;

if ft == 1

xPhys = sum(x(:)'.*H,2)./Hs;

elseif ft == 2

xTilde = sum(x(:)'.*H,2)./Hs;

xPhys = 1-exp(-beta*xTilde)+xTilde*exp(-beta);

end

thetaPhys = theta;

iH = ones(nelx*nely*(2*(ceil(rmin2/ab)-1)+1)^2,1);

jH = ones(size(iH));

sH = zeros(size(iH));

k = 0;

for i1 = 1:nelx

for j1 = 1:nely

e1 = (i1-1)*nely+j1;

for i2 = max(i1-(ceil(rmin2/ab)-1),1):min(i1+(ceil(rmin2/ab)-1),nelx)

for j2 = max(j1-(ceil(rmin2/ab)-1),1):min(j1+(ceil(rmin2/ab)-1),nely)

e2 = (i2-1)*nely+j2;

k = k+1;

iH(k) = e1;

jH(k) = e2;

d1=(i1-i2)*ab;

d2=(j2-j1)*ab;

sH(k) = max(0,rmin2-sqrt(d1^2+d2^2))*heaviside(3*pi/4-abs(theta(e1)-theta(e2)));

end

end

end

end

H2 = sparse(iH,jH,sH);

H2(PassDomain(:),:)=[];

H2(:,PassDomain(:))=[];

Hs2 = sum(H2,2);

if fj == 1

thetaPhys(ValidDomain1) = theta(ValidDomain1);

elseif fj==2

thetaPhys(ValidDomain1) = (H2*theta(ValidDomain1))./Hs2;

end

xPhys(PassDomain) = 10e-9;

x(PassDomain) = 10e-9;

loopbeta = 0;

loop = 0;

C=[];

Cp=[];

change = 1;

allxphyes=[];

m=1;

n1=size(ValidDomain(:),1);

n2=size(ValidDomain1(:),1);

n = n1+n2;

xold1 = repmat(0,n,1);

xold2 = xold1;

xmin = max([10e-9*ones(n1,1);thetamin*ones(n2,1)],[reshape(x(ValidDomain),n1,1)-move;reshape(theta(ValidDomain1),n2,1)-movetheta]);

xmax = min([ones(n1,1);thetamax*ones(n2,1)],[reshape(x(ValidDomain),n1,1)+move;reshape(theta(ValidDomain1),n2,1)+movetheta]);

low = xmin;

upp = xmax;

a0 = 1;

a = zeros(m,1);

c = 6000*ones(m,1);

d = zeros(m,1);

f0=0;

MaxItr=500;

changetheta=1;

allxphyes=[];

alltheatephyes=[];

while abs(change) > 0.00001 %||changemove(loop-1)> 0.00004%&& loop < MaxItr|| full(max(Fe(:)))>=1

n=nelx*nely;

loop = loop + 1;

loopbeta = loopbeta+1;

xs=thetaPhys;

ke=reshape(xPhys,n,1).^p1.*f(xs(:));

ke=ke';

sK = reshape(ke,64*n,1);

K = sparse(iK,jK,sK);

K = (K+K')/2;

U(freedofs) = K(freedofs,freedofs)\F(freedofs);

%% OBJECTIVE FUNCTION AND SENSITIVITY ANALYSIS

ke=f(xs(:));

ke=reshape(ke',8,8*n);

ke=mat2cell(ke,8,8*ones(1,n));

ke=ke';

ue=mat2cell(U(edofMat),ones(1,n),8);

ce = cellfun(@mtimes,ue,ke, 'UniformOutput',0);

ce=cell2mat(ce);

ue=cell2mat(ue);

ce=sum(ce.*ue,2);

singlece=reshape(reshape(xPhys,n,1).^p1.*ce,nely,nelx);

f0 = sum(singlece(:));

C(loop)=f0;

dcw = -p1*reshape(xPhys,n,1).^(p1-1).*ce;

dvw = ones(nely*nelx,1);

dc=zeros((nely+1),(nelx+1));

dv=zeros((nely+1),(nelx+1));

if ft==1

dc = sum((dcw.*H)./Hs);

dv = sum((dvw.*H)./Hs);

elseif ft == 2

dx = beta*exp(-beta*xTilde)+exp(-beta);

dc = sum((dcw.*H)./Hs.*dx);

dv = sum((dvw.*H)./Hs.*dx);

end

dke=f1(xs(:));

dke=reshape(dke',8,8*n);

dke=mat2cell(dke,8,8*ones(1,n));

ue=mat2cell(U(edofMat),ones(1,n),8);

ce1 = cellfun(@mtimes,ue,dke', 'UniformOutput',0);

ce1=cell2mat(ce1);

ue=cell2mat(ue);

ce1=sum(ce1.*ue,2);

dctheta = -reshape(xPhys,n,1).^p1.*ce1;

dctheta=reshape(dctheta,nely,nelx);

if fj==1

dctheta(ValidDomain1) =(H2*dctheta(ValidDomain1))./Hs2;

elseif fj==2

dctheta(ValidDomain1) = H2*(dctheta(ValidDomain1)./Hs2);

end

xval = [reshape(x(ValidDomain),n1,1);reshape(theta(ValidDomain1),n2,1)];

xmin = max([10e-9*ones(n1,1);thetamin*ones(n2,1)],[reshape(x(ValidDomain),n1,1)-move;reshape(theta(ValidDomain1),n2,1)-movetheta]);

xmax = min([ones(n1,1);thetamax*ones(n2,1)],[reshape(x(ValidDomain),n1,1)+move;reshape(theta(ValidDomain1),n2,1)+movetheta]);

f0val = f0;

df0dx = [reshape(dc(ValidDomain),n1,1);reshape(dctheta(ValidDomain1),n2,1)];

fval = [sum(xPhys(ValidDomain1))-volfrac*n1];

dfdx = [reshape(dv(ValidDomain),1,n1),zeros(1,n2)];

[xmma,~,~,~,~,~,~,~,~,low1,upp1] = ...

mmasub(m,n1+n2,loop,xval,xmin,xmax,xold1,xold2,f0val,df0dx,fval,dfdx,low,upp,a0,a,c,d);

xold2 = xold1;

xold1 = xval;

low = low1;

upp = upp1;

xold=x;

thetaold=theta;

x(ValidDomain)=xmma(1:n1,1);

theta(ValidDomain1)=xmma(1+n1:n1+n2,1);

if ft == 1

xPhys = sum(x(:)'.*H,2)./Hs;

elseif ft == 2

xTilde = sum(x(:)'.*H,2)./Hs;

xPhys = 1-exp(-beta*xTilde)+xTilde*exp(-beta);

end

iH = ones(nelx*nely*(2*(ceil(rmin2/ab)-1)+1)^2,1);

jH = ones(size(iH));

sH = zeros(size(iH));

k = 0;

for i1 = 1:nelx

for j1 = 1:nely

e1 = (i1-1)*nely+j1;

for i2 = max(i1-(ceil(rmin2/ab)-1),1):min(i1+(ceil(rmin2/ab)-1),nelx)

for j2 = max(j1-(ceil(rmin2/ab)-1),1):min(j1+(ceil(rmin2/ab)-1),nely)

e2 = (i2-1)*nely+j2;

k = k+1;

iH(k) = e1;

jH(k) = e2;

d1=(i1-i2)*ab;

d2=(j2-j1)*ab;

sH(k) = max(0,rmin2-sqrt(d1^2+d2^2))*heaviside(3*pi/4-abs(theta(e1)-theta(e2)));

end

end

end

end

H2 = sparse(iH,jH,sH);

H2(PassDomain(:),:)=[];

H2(:,PassDomain(:))=[];

Hs2 = sum(H2,2);

if fj == 1

thetaPhys(ValidDomain1) = theta(ValidDomain1);

elseif fj==2

thetaPhys(ValidDomain1) = (H2*theta(ValidDomain1))./Hs2;

end

Mnd=sum(4*xPhys(:).*(1-xPhys(:)))/(nelx*nely);

if loop==1

change=1;

else

change = abs(C(loop)-C(loop-1))/C(loop);

end

changex = max(max(abs(x(:)-xold(:))));

changetheta=max(max(abs(theta(:)-thetaold(:))));

disp([' It.: ' sprintf('%4i',loop) ' Obj.: ' sprintf('%10.4f',f0) ...

' Vol.: ' sprintf('%6.3f',sum(sum(xPhys))/(n1)) ' Mnd.: ' sprintf('%6.3f',Mnd)...

' ch.: ' sprintf('%10.5f',change) ...

' chx.: ' sprintf('%6.3f',changex) ' chtheta.: ' sprintf('%6.3f',full(changetheta))])

if p1 < 5 && change <= 0.005

p1 = p1+0.5;

fprintf('Parameter p1 increased to %g.\n',p1);

end

% if ft == 2 && beta < 50 &&(loopbeta >= 50 || change <= 0.0006)

if ft == 2 && beta < 50 &&change <= 0.005

%beta = 2*beta;

beta = beta+1;

loopbeta = 0;

change = 1;

fprintf('Parameter beta increased to %g.\n',beta);

end

% beta=min(50,beta+1.1^loopbeta)

xPhys=reshape(xPhys,nely,nelx);

allxphyes{loop}=xPhys;

alltheatephyes{loop}=thetaPhys;

end

%-------------------------------------------------------

% This is the file elementfea.m

%

function [KE,dKE,D0,trsT,dtrsT,B_]=elementfea(E1,E2,G12,NU12,NU21,a)

syms x y theta

a=a/2;

x1=-a;

y1=-a;

x2=a;

y2=-a;

x3=a;

y3=a;

x4=-a;

y4=a;

x0=(x1+x2)/2;

y0=(y1+y4)/2;

a=(x2-x1)/2;

b=(y3-y2)/2;

xi=(x-x0)/a;

eta =(y-y0)/b;

N1=(1-xi)*(1-eta)/4;N2=(1+xi)*(1-eta)/4;N3=(1+xi)*(1+eta)/4;N4=(1-xi)*(1+eta)/4;

%% 对xy求导

N1x=diff(N1,x);N2x=diff(N2,x);N3x=diff(N3,x);N4x=diff(N4,x);

N1y=diff(N1,y);N2y=diff(N2,y);N3y=diff(N3,y);N4y=diff(N4,y);

B=[N1x 0 N2x 0 N3x 0 N4x 0;

0 N1y 0 N2y 0 N3y 0 N4y;

N1y N1x N2y N2x N3y N3x N4y N4x];

D0 = [E1/(1-NU12*NU21),E1*NU21/(1-NU12*NU21), 0 ; E1*NU21/(1-NU12*NU21), E2/(1-NU12*NU21), 0 ; 0, 0, G12];

T=[cos(theta)^2 sin(theta)^2 -2*cos(theta)*sin(theta);sin(theta)^2 cos(theta)^2 2*cos(theta)*sin(theta);cos(theta)*sin(theta) -cos(theta)*sin(theta) cos(theta)^2-sin(theta)^2];

D=T*D0*T.';

dD=diff(D,theta);

KE=int(int(B.'*D*B,x,x1,x2),y,y1,y4);

dKE=int(int(B.'*dD*B,x,x1,x2),y,y1,y4);

trsT=T.';

dtrsT=diff(trsT,theta);

B_=subs(B,{x,y},{(x1+x2)/2,(y1+y4)/2});

end

%-------------------------------------------------------

% This is the file mmasub.m

%

function [xmma,ymma,zmma,lam,xsi,eta,mu,zet,s,low,upp] = ...

mmasub(m,n,iter,xval,xmin,xmax,xold1,xold2, ...

f0val,df0dx,fval,dfdx,low,upp,a0,a,c,d)

%

% Version September 2007 (and a small change August 2008)

%

% Krister Svanberg <krille@math.kth.se>

% Department of Mathematics, SE-10044 Stockholm, Sweden.

%

% This function mmasub performs one MMA-iteration, aimed at

% solving the nonlinear programming problem:

%

% Minimize f_0(x) + a_0*z + sum( c_i*y_i + 0.5*d_i*(y_i)^2 )

% subject to f_i(x) - a_i*z - y_i <= 0, i = 1,...,m

% xmin_j <= x_j <= xmax_j, j = 1,...,n

% z >= 0, y_i >= 0, i = 1,...,m

%*** INPUT:

%

% m = The number of general constraints.

% n = The number of variables x_j.

% iter = Current iteration number ( =1 the first time mmasub is called).

% xval = Column vector with the current values of the variables x_j.

% xmin = Column vector with the lower bounds for the variables x_j.

% xmax = Column vector with the upper bounds for the variables x_j.

% xold1 = xval, one iteration ago (provided that iter>1).

% xold2 = xval, two iterations ago (provided that iter>2).

% f0val = The value of the objective function f_0 at xval.

% df0dx = Column vector with the derivatives of the objective function

% f_0 with respect to the variables x_j, calculated at xval.

% fval = Column vector with the values of the constraint functions f_i,

% calculated at xval.

% dfdx = (m x n)-matrix with the derivatives of the constraint functions

% f_i with respect to the variables x_j, calculated at xval.

% dfdx(i,j) = the derivative of f_i with respect to x_j.

% low = Column vector with the lower asymptotes from the previous

% iteration (provided that iter>1).

% upp = Column vector with the upper asymptotes from the previous

% iteration (provided that iter>1).

% a0 = The constants a_0 in the term a_0*z.

% a = Column vector with the constants a_i in the terms a_i*z.

% c = Column vector with the constants c_i in the terms c_i*y_i.

% d = Column vector with the constants d_i in the terms 0.5*d_i*(y_i)^2.

%

%*** OUTPUT:

%

% xmma = Column vector with the optimal values of the variables x_j

% in the current MMA subproblem.

% ymma = Column vector with the optimal values of the variables y_i

% in the current MMA subproblem.

% zmma = Scalar with the optimal value of the variable z

% in the current MMA subproblem.

% lam = Lagrange multipliers for the m general MMA constraints.

% xsi = Lagrange multipliers for the n constraints alfa_j - x_j <= 0.

% eta = Lagrange multipliers for the n constraints x_j - beta_j <= 0.

% mu = Lagrange multipliers for the m constraints -y_i <= 0.

% zet = Lagrange multiplier for the single constraint -z <= 0.

% s = Slack variables for the m general MMA constraints.

% low = Column vector with the lower asymptotes, calculated and used

% in the current MMA subproblem.

% upp = Column vector with the upper asymptotes, calculated and used

% in the current MMA subproblem.

%

%epsimin = sqrt(m+n)*10^(-9);

epsimin = 10^(-8);

% epsimin = 10^(-10);

raa0 = 0.01;

if iter<3

albefa = 0.6;

else

albefa = 0.4;

end

% asyinit = 0.10;

% asyincr = 0.8;

% asydecr = 0.6;

% asyinit = 0.50;

% asyincr = 1.2;

% asydecr = 0.7;

asyinit = 0.5;%%取值范围0-1开区间，看mmagcmma这个pdf

asyincr = 1.5;

asydecr = 0.8;

eeen = ones(n,1);

eeem = ones(m,1);

zeron = zeros(n,1);

% Calculation of the asymptotes low and upp :

if iter < 2.5

low = xval - asyinit*(xmax-xmin);

upp = xval + asyinit*(xmax-xmin);

else

zzz = (xval-xold1).*(xold1-xold2);

factor = eeen;

factor(find(zzz > 0)) = asyincr;

factor(find(zzz < 0)) = asydecr;

low = xval - factor.*(xold1 - low);

upp = xval + factor.*(upp - xold1);

lowmin = xval - 10*(xmax-xmin);

lowmax = xval - 0.01*(xmax-xmin);

uppmin = xval + 0.01*(xmax-xmin);

uppmax = xval + 10*(xmax-xmin);

% if iter>20&&iter<100

% lowmin = xval - 0.02*(xmax-xmin);

% lowmax = xval - 0.01*(xmax-xmin);

% uppmin = xval + 0.01*(xmax-xmin);

% uppmax = xval + 0.02*(xmax-xmin);

% end

low = max(low,lowmin);

low = min(low,lowmax);

upp = min(upp,uppmax);

upp = max(upp,uppmin);

end

% Calculation of the bounds alfa and beta :

zzz = low + albefa*(xval-low);

alfa = max(zzz,xmin);

zzz = upp - albefa*(upp-xval);

beta = min(zzz,xmax);

% Calculations of p0, q0, P, Q and b.

xmami = xmax-xmin;

xmamieps = 0.00001*eeen;

xmami = max(xmami,xmamieps);

xmamiinv = eeen./xmami;

ux1 = upp-xval;

ux2 = ux1.*ux1;

xl1 = xval-low;

xl2 = xl1.*xl1;

uxinv = eeen./ux1;

xlinv = eeen./xl1;

%

p0 = zeron;

q0 = zeron;

% p0 = max(df0dx,0);

% q0 = max(-df0dx,0);

p0(find(df0dx > 0)) = df0dx(find(df0dx > 0));

q0(find(df0dx < 0)) = -df0dx(find(df0dx < 0));

pq0 = 0.001*(p0 + q0) + raa0*xmamiinv;

p0 = p0 + pq0;

q0 = q0 + pq0;

p0 = p0.*ux2;

q0 = q0.*xl2;

%

% P = sparse(m,n);

% Q = sparse(m,n);

P = zeros(m,n);

Q = zeros(m,n);

% P = max(dfdx,0);

% Q = max(-dfdx,0);

P(find(dfdx > 0)) = dfdx(find(dfdx > 0));

Q(find(dfdx < 0)) = -dfdx(find(dfdx < 0));

PQ = 0.001*(P + Q) + raa0*eeem*xmamiinv';

P = P + PQ;

Q = Q + PQ;

P = P * spdiags(ux2,0,n,n);

Q = Q * spdiags(xl2,0,n,n);

b = P*uxinv + Q*xlinv - fval ;

%

%%% Solving the subproblem by a primal-dual Newton method

[xmma,ymma,zmma,lam,xsi,eta,mu,zet,s] = ...

subsolv(m,n,epsimin,low,upp,alfa,beta,p0,q0,P,Q,a0,a,b,c,d);

% This is the file subsolv.m

%

function [xmma,ymma,zmma,lamma,xsimma,etamma,mumma,zetmma,smma] = ...

subsolv(m,n,epsimin,low,upp,alfa,beta,p0,q0,P,Q,a0,a,b,c,d);

%

% Written in May 1999 by

% Krister Svanberg <krille@math.kth.se>

% Department of Mathematics

% SE-10044 Stockholm, Sweden.

%

% This function subsolv solves the MMA subproblem:

%

% minimize SUM[ p0j/(uppj-xj) + q0j/(xj-lowj) ] + a0*z +

% + SUM[ ci*yi + 0.5*di*(yi)^2 ],

%

% subject to SUM[ pij/(uppj-xj) + qij/(xj-lowj) ] - ai*z - yi <= bi,

% alfaj <= xj <= betaj, yi >= 0, z >= 0.

%

% Input: m, n, low, upp, alfa, beta, p0, q0, P, Q, a0, a, b, c, d.

% Output: xmma,ymma,zmma, slack variables and Lagrange multiplers.

%

een = ones(n,1);

eem = ones(m,1);

epsi = 1;

epsvecn = epsi*een;

epsvecm = epsi*eem;

x = 0.5*(alfa+beta);

y = eem;

z = 1;

lam = eem;

xsi = een./(x-alfa);

xsi = max(xsi,een);

eta = een./(beta-x);

eta = max(eta,een);

mu = max(eem,0.5*c);

zet = 1;

s = eem;

itera = 0;

while epsi > epsimin

epsvecn = epsi*een;

epsvecm = epsi*eem;

ux1 = upp-x;

xl1 = x-low;

ux2 = ux1.*ux1;

xl2 = xl1.*xl1;

uxinv1 = een./ux1;

xlinv1 = een./xl1;

plam = p0 + P'*lam ;

qlam = q0 + Q'*lam ;

gvec = P*uxinv1 + Q*xlinv1;

dpsidx = plam./ux2 - qlam./xl2 ;

rex = dpsidx - xsi + eta;

rey = c + d.*y - mu - lam;

rez = a0 - zet - a'*lam;

relam = gvec - a*z - y + s - b;

rexsi = xsi.*(x-alfa) - epsvecn;

reeta = eta.*(beta-x) - epsvecn;

remu = mu.*y - epsvecm;

rezet = zet*z - epsi;

res = lam.*s - epsvecm;

residu1 = [rex' rey' rez]';

residu2 = [relam' rexsi' reeta' remu' rezet res']';

residu = [residu1' residu2']';

residunorm = sqrt(residu'*residu);

residumax = max(abs(residu));

ittt = 0;

while residumax > 0.9*epsi & ittt < 100

ittt=ittt + 1;

itera=itera + 1;

ux1 = upp-x;

xl1 = x-low;

ux2 = ux1.*ux1;

xl2 = xl1.*xl1;

ux3 = ux1.*ux2;

xl3 = xl1.*xl2;

uxinv1 = een./ux1;

xlinv1 = een./xl1;

uxinv2 = een./ux2;

xlinv2 = een./xl2;

plam = p0 + P'*lam ;

qlam = q0 + Q'*lam ;

gvec = P*uxinv1 + Q*xlinv1;

GG = P*spdiags(uxinv2,0,n,n) - Q*spdiags(xlinv2,0,n,n);

dpsidx = plam./ux2 - qlam./xl2 ;

delx = dpsidx - epsvecn./(x-alfa) + epsvecn./(beta-x);

dely = c + d.*y - lam - epsvecm./y;

delz = a0 - a'*lam - epsi/z;

dellam = gvec - a*z - y - b + epsvecm./lam;

diagx = plam./ux3 + qlam./xl3;

diagx = 2*diagx + xsi./(x-alfa) + eta./(beta-x);

diagxinv = een./diagx;

diagy = d + mu./y;

diagyinv = eem./diagy;

diaglam = s./lam;

diaglamyi = diaglam+diagyinv;

if m < n

blam = dellam + dely./diagy - GG*(delx./diagx);

bb = [blam' delz]';

Alam = spdiags(diaglamyi,0,m,m) + GG*spdiags(diagxinv,0,n,n)*GG';

AA = [Alam a

a' -zet/z ];

solut = AA\bb;

dlam = solut(1:m);

dz = solut(m+1);

dx = -delx./diagx - (GG'*dlam)./diagx;

else

diaglamyiinv = eem./diaglamyi;

dellamyi = dellam + dely./diagy;

Axx = spdiags(diagx,0,n,n) + GG'*spdiags(diaglamyiinv,0,m,m)*GG;

azz = zet/z + a'*(a./diaglamyi);

axz = -GG'*(a./diaglamyi);

bx = delx + GG'*(dellamyi./diaglamyi);

bz = delz - a'*(dellamyi./diaglamyi);

AA = [Axx axz

axz' azz ];

bb = [-bx' -bz]';

solut = AA\bb;

dx = solut(1:n);

dz = solut(n+1);

dlam = (GG*dx)./diaglamyi - dz*(a./diaglamyi) + dellamyi./diaglamyi;

end

dy = -dely./diagy + dlam./diagy;

dxsi = -xsi + epsvecn./(x-alfa) - (xsi.*dx)./(x-alfa);

deta = -eta + epsvecn./(beta-x) + (eta.*dx)./(beta-x);

dmu = -mu + epsvecm./y - (mu.*dy)./y;

dzet = -zet + epsi/z - zet*dz/z;

ds = -s + epsvecm./lam - (s.*dlam)./lam;

xx = [ y' z lam' xsi' eta' mu' zet s']';

dxx = [dy' dz dlam' dxsi' deta' dmu' dzet ds']';

stepxx = -1.01*dxx./xx;

stmxx = max(stepxx);

stepalfa = -1.01*dx./(x-alfa);

stmalfa = max(stepalfa);

stepbeta = 1.01*dx./(beta-x);

stmbeta = max(stepbeta);

stmalbe = max(stmalfa,stmbeta);

stmalbexx = max(stmalbe,stmxx);

stminv = max(stmalbexx,1);

steg = 1/stminv;

xold = x;

yold = y;

zold = z;

lamold = lam;

xsiold = xsi;

etaold = eta;

muold = mu;

zetold = zet;

sold = s;

itto = 0;

resinew = 2*residunorm;

while resinew > residunorm & itto < 50

itto = itto+1;

x = xold + steg*dx;

y = yold + steg*dy;

z = zold + steg*dz;

lam = lamold + steg*dlam;

xsi = xsiold + steg*dxsi;

eta = etaold + steg*deta;

mu = muold + steg*dmu;

zet = zetold + steg*dzet;

s = sold + steg*ds;

ux1 = upp-x;

xl1 = x-low;

ux2 = ux1.*ux1;

xl2 = xl1.*xl1;

uxinv1 = een./ux1;

xlinv1 = een./xl1;

plam = p0 + P'*lam ;

qlam = q0 + Q'*lam ;

gvec = P*uxinv1 + Q*xlinv1;

dpsidx = plam./ux2 - qlam./xl2 ;

rex = dpsidx - xsi + eta;

rey = c + d.*y - mu - lam;

rez = a0 - zet - a'*lam;

relam = gvec - a*z - y + s - b;

rexsi = xsi.*(x-alfa) - epsvecn;

reeta = eta.*(beta-x) - epsvecn;

remu = mu.*y - epsvecm;

rezet = zet*z - epsi;

res = lam.*s - epsvecm;

residu1 = [rex' rey' rez]';

residu2 = [relam' rexsi' reeta' remu' rezet res']';

residu = [residu1' residu2']';

resinew = sqrt(residu'*residu);

steg = steg/2;

end

residunorm=resinew;

residumax = max(abs(residu));

steg = 2*steg;

end

epsi = 0.1*epsi;

end

xmma = x;

ymma = y;

zmma = z;

lamma = lam;

xsimma = xsi;

etamma = eta;

mumma = mu;

zetmma = zet;

smma = s;
